# Effect of the combined intervention of low‐FODMAPs diet and probiotics on IBS symptoms in Western China: A randomized controlled trial

**DOI:** 10.1002/fsn3.4057

**Published:** 2024-02-29

**Authors:** Yingying Liu, Di Jin, Tian He, Xinyi Liao, Limei Shao, Lei Shi, Ling Liu

**Affiliations:** ^1^ Department of Gastroenterology and Hepatology West China Hospital, Sichuan University Chengdu China; ^2^ Department of Urology West China Hospital of Sichuan University Chengdu China; ^3^ Department of Clinical Nutrition West China Hospital, Sichuan University Chengdu China

**Keywords:** irritable bowel syndrome, low‐FODMAPs diet, probiotics, Western China

## Abstract

The effect of low‐FODMAPs diet on irritable bowel syndrome (IBS) in Western China has not been reported. We aimed to investigate the effect of low‐FODMAPs diet on IBS patients in the area and whether low‐FODMAPs diet‐induced alterations of microbiota could be improved through probiotics. IBS patients were randomized to the control group, low‐FODMAPs diet group, probiotics group, or combined group. IBS Symptom Severity Score questionnaire (IBS‐SSS) and IBS Quality of Life Score questionnaire (IBS‐QOL) were completed at baseline, 2 and 4 weeks to evaluate the severity of symptoms. Fresh feces were collected for analyses of gut microbiota and short‐chain fatty acids at baseline and 4 weeks after intervention. Seventy‐three patients were included in the per protocol analysis. After intervention, there was significant improvement in IBS‐SSS in the low‐FODMAPs group (37.5%, 44.2%), probiotics group (51.4%, 62.0%), and combined group (34.1%, 40.4%) at both 2 weeks and 4 weeks, compared with the baseline (*p* < .05). In the low‐FODMAPs group, the abundance of several microbiota (*Lachnoclostridium*, *Enterococcus*, etc.) was significantly decreased. Furthermore, after the supplementation of probiotics in the combined group, the abundance of Genus_*Ruminococcus*, *Coprococcus*, *Acidaminococcus*, *Ruminiclostridium*, *Akkermansia*, *Eggerthella*, and *Oxalobacter* was significantly increased, which was associated with the improvements of symptoms score in the *Pearson* correlation analysis. Our study confirmed the effectiveness and safety of short‐term low‐FODMAPs diet in IBS symptoms based on the Chinese diet in Western China. The combination of low‐FODMAPs and probiotics plays a beneficial role in gut microbiota in IBS.

## Graphical abstract

Low‐FODMAPs diet and low‐FODMAPs diet combined with probiotics could significantly relieve the symptoms of IBS patients. The addition of probiotics increased the abundance of several gut microbiota, including *Ruminococcus*, *Coprococcus, Acidaminococcus* et al. *Pearson* correlation analysis confirmed that alterations of the microbiota induced by probiotics were significantly associated with the improvements in IBS symptoms.

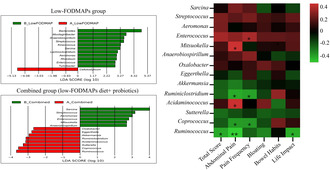

## 1 INTRODUCTION

Irritable bowel syndrome (IBS) is a distressing functional gastrointestinal disorder of long duration that affects approximately 1 in 10 population globally (Black & Ford, 2020). The main symptoms of patients with IBS are abdominal pain, bloating, and changes in bowel habits (e.g., diarrhea, constipation, or a combination of both) (Alammar & Stein, 2019). Multiple management have been used in clinic for the treatment of IBS, including pharmacological, dietary, psychological, and other behavioral approaches. Among them, dietary intervention (Lacy et al., 2021) and probiotics treatment (Simon et al., 2021) have been gradually applied to the clinical treatment.

Low‐FODMAPs diet, which restricts foods high in oligosaccharides, disaccharides, monosaccharides, and polyols, is one of the widely adopted dietary treatments (Black et al., 2022). The low‐FODMAPs diet was superior to other types of dietary interventions for IBS symptoms (Black et al., 2022). A beneficial short‐term effect of low‐FODMAPs diet was confirmed in a majority of IBS patients, through the alleviation of IBS symptoms, in particular with bloating, flatulence, diarrhea, and global symptoms (Schumann et al., 2018; van Lanen et al., 2021). Although there are enough studies to confirm that a low‐FODMAPs diet relives IBS symptoms, most of these studies are based on a western dietary background. The significant differences between Chinese and western diets cannot be ignored. Therefore, the efficacy of low‐FODMAPs diet based on the Chinese diet for the treatment of IBS needs to be further verified (Zhang et al., 2021). To our knowledge, no RCT has been reported on the effectiveness of low‐FODMAPs diet on IBS patients in Western China, where the main dietary structure is Sichuan cuisine, one of the four major cuisines in China. Sichuan cuisine is famous for its pungency and spice flavor, which results from the liberal use of strong pepper, chili, garlic, thick broad‐bean sauce, and other unique Sichuan ingredients, such as ginger and mustard. Most of these ingredients have high FODMAPs content. Therefore, it is of interest to investigate the effect of a low‐FODMAPs diet on IBS patients in the area.

The gut microbiota integrates with diet, genetic, and immunity signals to regulate the host's metabolism, immunity, and inflammation (Hou et al., 2022). Substantial evidence indicates that dysbiosis of the gut microbiota is an important feature of IBS, such as the decreased α‐diversity and the changes in *Firmicutes* to *Bacteroidetes* ratio, and it might be involved in its pathogenesis (Duan et al., 2019).

FODMAPs are substrates of bacterial fermentation, which produce short‐chain fatty acids (SCFAs). SCFAs are beneficial to the gut through a variety of mechanisms, such as regulating Tregs and inhibiting histone deacetylases (Kim, 2021; Makki et al., 2018). Nevertheless, the adverse effects of low‐FODMAPs diet on the gut microbiota reported in previous studies cannot be ignored (McIntosh et al., 2017; Vervier et al., 2022). Except for the decrease in the abundance of *Bifidobacteria* after restriction of FODMAPs (So et al., 2022), most of the related data were controversial. Co‐administration of *Bifidobacteria* could restored numbers of *Bifidobacterium* species in low‐FODMAPs diet, but no significant alleviation of IBS symptoms was reported (Staudacher et al., 2017). Therefore, we wondered whether the combination of probiotics with multiple species, including *Bifidobacterium*, *Lactobacillus bulgaricus*, and *Streptococcus thermophilus* may further alleviate IBS symptoms under low‐FODMAPs diet.

In order to address the questions we mentioned above, we designed this prospective trial with two main objectives. Firstly, we aimed to investigate the effect of the low‐FODMAPs diet on IBS patients, based on the Western Chinese diet. Second, we investigated whether the alterations in the microbiota induced by low‐FODMAPs diet could be improved through supplementation of probiotics.

## 2 MATERIALS AND METHODS

### 2.1 Study design and participants

This randomized controlled prospective trial was conducted according to the guidelines established in the Declaration of Helsinki. All procedures were approved by the West China Hospital Human Research Ethics Committee and registered in the Chinese clinical trial registry (www.chictr.org.cn, ChiCTR1900026666). This was a single‐center study conducted at the Gastrointestinal (GI) outpatient center of West China Hospital, a tertiary hospital, Chengdu, China. We got the informed consent from all participants. The details of the informed consent were provided in the Supplementary Material.

An independent researcher, who was not involved in screening or recruitment, conducted a simple random grouping using a random number table. Randomized numbers were sealed in opaque envelopes sequentially and divided by 4. The remainder of 0, 1, 2, and 3 were assigned to the conventional diet (Control group), lower dietary intake of FODMAPs (low‐FODMAPs group), supplementation of probiotics with conventional diet (Probiotics group), or combination of low‐FODMAPs and probiotics (Combined group), respectively. Researchers responsible for enrolment, randomization, telephone follow‐up, data processing, questionnaire analysis, microbiota analysis, and statistical analysis were unaware of the randomization groups. Patients were blinded to the dietary intervention.

From 2018 to 2021, patients with IBS aged 18–65 years who met the Rome IV criteria (Drossman & Hasler, 2016) were recruited from the outpatient department. Patients with heart, lung, kidney, or liver dysfunction; brain diseases; or metabolic diseases, including hypothyroidism, hyperthyroidism, or diabetes, were excluded by history taking, questionnaire survey, biochemical test, abdominal ultrasonography, CT scan, gastroduodenoscopy, or colonoscopy screening. In addition, those with the following conditions were not eligible to participate in our study: previous gastrointestinal resection, pregnancy, use of probiotics or prebiotics, dietary intervention, use of prokinetic drugs, or bowel preparation within 4 weeks prior to the study. Ingestion of other probiotics, prebiotics, or antibiotics by patients during the study was not permitted. Once a violation of the protocol occurred during the study, the patient will be withdrawn and excluded from the per protocol (PP) analysis.

### 2.2 Trial protocol

**FIGURE 1:**
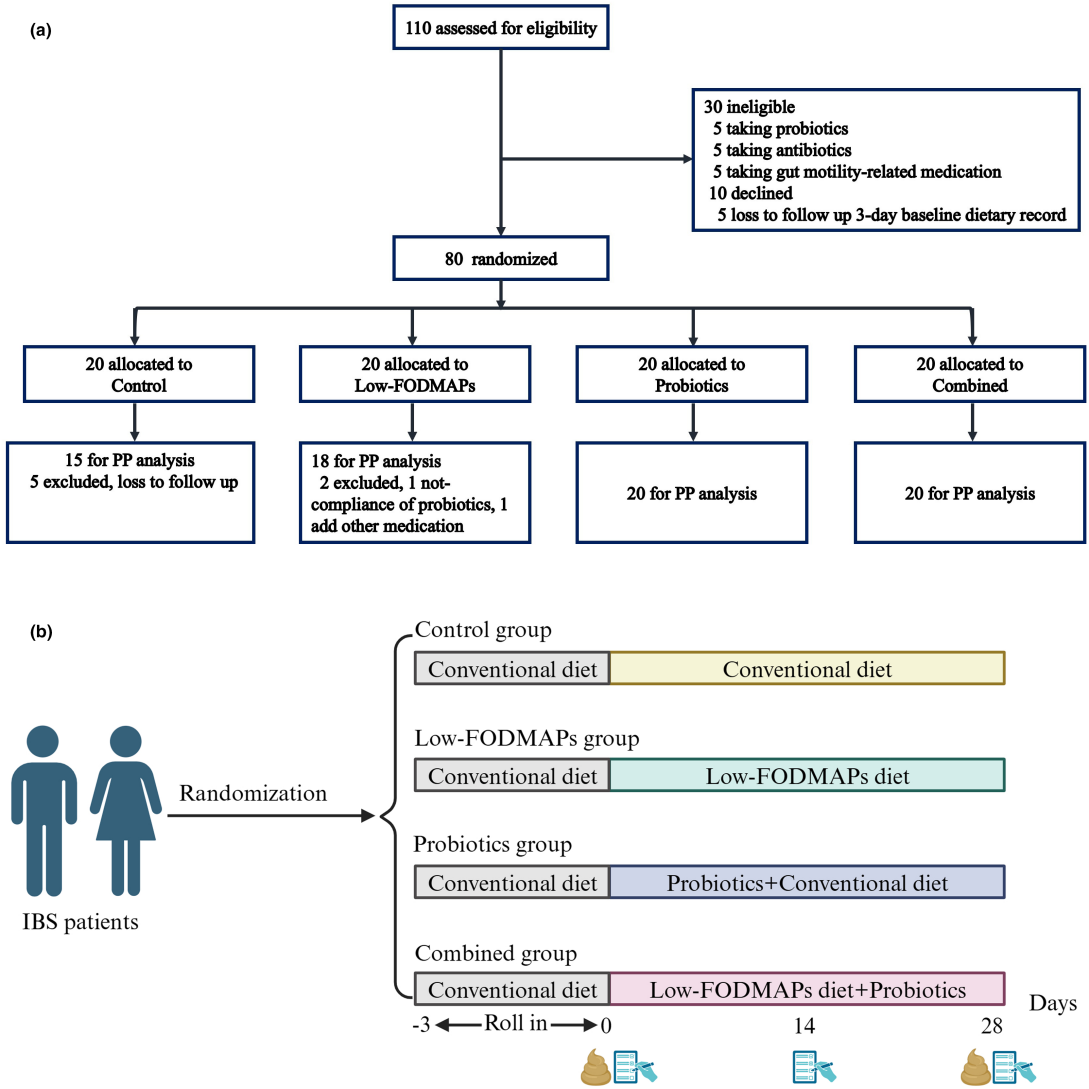
Trial procedure and experimental flow chart. (a) Procedures for inclusion and grouping of irritable bowel syndrome patients. (b) The experimental flow chart: A 4‐week intervention was conducted for the four groups of subjects, fresh stool was collected on day 0 and day 28, and questionnaires were completed on day 2, week 2 (day 14), and week 4 (day 28).

Our RCT was conducted in accordance with the CONSORT guidelines (Supplementary Material). Patients who were eligible for and willing to participate in the study were recruited and randomly allocated to the control group, low‐FODMAPs group, probiotics group, or combined group, with at least 15 patients per group included in the PP analysis (Figure 1a). Before the intervention of 3 days, all patients were advised to maintain a habitual diet and to send pictures of all the food (including fruit and soft drinks) eaten daily to the designated researcher through an online communication platform. It was considered a 3‐day run‐in period. The researcher will categorize foods as a low‐ or high‐FODMAPs diet based on the pictures. Daily stool characteristics and gastrointestinal symptoms were also recorded. After the start of the trial, participants in different groups were required to adjust their diet or take tablets as needed, take pictures, and upload them.

Patients in the low‐FODMAPs group are advised to limit their consumption of foods high in FODMAPs, including cereals (large amounts of wheat, rye, such as noodles, bread, crackers, biscuits, steamed rye flour, pasta, and chrysanthemum starch); dairy (milk, goat milk, ice cream, yogurt, and raw cheese); legumes (chickpeas, kidney beans, lentils, and soybeans); vegetables (e.g., ginger, asparagus, broccoli, cabbage, fennel, garlic, leek, okra, onion, green onion, chicory, dandelion, inulin, cauliflower, green pepper, mushroom, sweet corn); fruit (e.g., apple, mango, pear, watermelon, apricot, avocado, blackberry, cherry, nut); others (e.g., sweeteners: such as fructose, corn syrup, juice, honey, sorbitol, mannitol, maltitol, and xylitol). This dietary recommendation is consistent with those used in previous studies (Barrett et al., 2010; Ong et al., 2010; Zhang et al., 2021). The average daily intake of FODMAPs before and after intervention was calculated based on the excess fructose, lactose, mannitol, sorbitol, galacto‐oligosaccharide (GOS), and total fructan contents of various foods including fruits, snacks, dairy products, nuts, and other foods obtained in previous studies (Biesiekierski et al., 2011; Chumpitazi et al., 2018; Muir et al., 2007, 2009; Prichard et al., 2016; Tuck et al., 2018; Varney et al., 2017).

The probiotics group was supplied with a probiotic chewable tablet, named *Golden Bifidobacteria* (Jing Shuangqi) (Inner Mongolia *Bifidobacteria* Pharmaceutical Industry, Wanze Group, Shenzhen, China), which is a commonly used clinical drug in China, containing *Bifidobacterium* (not less than 1.0 × 10^7^CFU per gram), *Lactobacillus bulgaricus* (not less than 1.0 × 10^6^ CFU/g), and *Streptococcus thermophilus* (not less than 1.0 × 10^6^ CFU/g) with the dosage of 0.5 g/tablet. The dosage of *Golden Bifidobacteria* for patient's intervention was 2 g/time, 3 times/day. Tablets were kept in a refrigerator at 4°C to maintain probiotic activity.

Patients in the control group and probiotics group continued their habitual Chinese diet, followed the recommendation of conventional dietary, including regular meals, adequate water intake, decreased intake of fat, alcohol, caffeine, spicy foods, and the foods that may aggravate intestinal symptoms. All recommendations were given by the same experienced dietician, who was blinded to the data analysis.

The intervention time lasted for 4 weeks. Both questionnaires of the IBS Symptom Severity Score (IBS‐SSS) (Francis et al., 1997) and the IBS Quality of Life Score (IBS‐QOL) (Patrick et al., 1998) were completed at 2 weeks and 4 weeks by face‐to‐face interview at the outpatient center (Figure 1b). The contents of these questionnaires are presented in the Supplementary Material.

### 2.3 16S rDNA Illumina sequencing and bioinformatic analyses

Fresh feces within 1 hour of passage were collected at preintervention baseline and 4 weeks after intervention, and specimens were stored in −150°C frozen phosphate buffer until further processing. The bacterial genomic DNA was extracted from all the samples. A DNA library was constructed by PCR amplification of the16S rDNA V3‐V4 region and sequenced on the Illumina MiSeq Sequencing platform, performed by We Healthy Gene Technology Co (Shenzhen, Guangzhou, China). We performed bioinformatics analyses as described previously (Zhang et al., 2018). Briefly, the Quantitative Insights Into Microbial Ecology was used to calculate the α‐diversity and β‐diversity. As mentioned in our previous study, we used linear discriminant analysis (LDA) to compare and display the significant differences between baseline and after 4 weeks of intervention (Jiao et al., 2020; Zhang et al., 2018).

### 2.4 Detection of SCFAs in the feces

1 g fecal sample was collected and filtered through a 0.45 μm membrane. The filtered samples were assayed according to the methods of previous studies (Jiao et al., 2020).

### 2.5 Outcomes

The primary outcome was the IBS Symptom Severity Score (IBS‐SSS) at the 4 weeks. The secondary outcomes were IBS Quality of Life Score (IBS‐QOL), and changes in the microbiota and SCFAs at the 4 weeks. Adverse events were recorded during daily online communication and follow‐up visits.

### 2.6 Statistical analysis

As this was an exploratory study based on previous findings (Rej et al., 2022; Staudacher et al., 2021; Wilson et al., 2023), the remission rates of IBS‐SSS in the combined group, Probiotic group, low‐FODMAPs group, and Control group were initially set to be 80%, 60%, 50%, and 20%, respectively. Assuming a power (1 − *β*) of 0.80, a two‐sided significance level (*α*) of 0.05, and a dropout rate of 10%. A minimum of 15 patients were included in each group, except for dropout subjects. PP analysis was performed using data from completed patients who did not violate the protocol. The data are presented as the mean ± SD or n (%) for demographic data. *Student's t* test or the *Wilcoxon rank sum* test was used for comparisons between two groups; *ANOVA*, among four groups; or the *Mann–Whitney* test was performed for continuous variables. The *Chi‐square* test and *Fisher's exact* test were performed for categorical variables. To explore the association between gut microbiota and IBS symptoms, *Pearson* correlation analysis was used. A two‐sided *p* value less than .05 was considered to indicate statistical significance. Data analysis was performed using SPSS software (SPSS Statistics, version 19.0, IBM Corp., Armonk, NY, USA) and Graphpad Prism (version 8.0.0, Graphpad software, SanDiego, California USA).

## 3 RESULTS

We recruited 110 IBS patients, of whom 30 were ineligible and were removed from the trial. The remaining 80 patients were randomized to the control, low‐FODMAPs, probiotics, or combined group, with 20 patients per group (Figure 1a). In this study, 7 patients were withdrawn from the trial, in which 5 patients who was lost to follow in the control group and 2 patients in the low‐FODMAPs group were not compliant with probiotics and added other medications. All 73 randomized patients, including 15 patients in the control group, 18 patients in the low‐FODMAPs group, and 20 patients in both the probiotics group and the combined group, were included in the PP analysis. No severe adverse effects were observed from the intervention in this study.

The baseline characteristics of patients were comparable for gender, age, and types of IBS subgroups (Table 1). All patients were Chinese. The composition of diarrhea, constipation, and mix subtypes was comparable among the 4 groups and 58.9% of the recruited patients were in the diarrhea subgroup.

**TABLE 1:** Baseline characteristics of irritable bowel syndrome patients.

	Control	Low‐FODMAPs	Probiotics	Combined
Numbers	15	18	20	20
Gender (male/ female)	8/7	12/6	10/10	10/10
Age (mean ± SD)	35.0 ± 9.8	34.9 ± 12.5	39.1 ± 9.5	40.5 ± 11.0
IBS subtype (diarrhea/constipation/mix)	10/3/2	10/3/5	11/5/4	12/2/6

### 3.1 Low‐FODMAPs diet intervention

All patients with the low‐FODMAPs intervention lasted for 4 weeks. The changes in low‐FODMAPs intake before and after diet intervention are shown in Table 2. Before intervention, the average types of Chinese diet consumed by the recruited patients were 30.2 ± 6.1, in which the types of low‐FODMAPs food were 23.7 ± 4.1 and high‐FODMAPs food were 6.5 ± 2.3, with 78.7 ± 4.0% of low‐FODMAPs types of food. After low‐FODMAPs intervention, the average types of food were 23.9 ± 5.2, in which the composition of high‐FODMAPs ingredients was decreased significantly (2.3 ± 1.2, *p* < .05) and the percentage of low‐FODMAPs was increased significantly (90.1 ± 5.1%) than that in the non‐FODMAPs intervention group (*p* < .05). After limiting low‐FODMAPs, the FODMAPs content in the daily food of the low‐FODMAPs group and the combined group decreased significantly, compared with that at baseline (*p* < .05, Table 3). There was no significant change in energy, protein, fat, carbohydrate, or dietary fiber in these two groups before or after dietary intervention, indicating that our dietary intervention was successful.

**TABLE 2:** Type changes of Low‐FODMAPs in the diet.

Types of food	Predietary intervention	Low‐FODMAPs
Total	30.2 ± 6.1	23.9 ± 5.2
Low‐FODMAPs	23.7 ± 4.1	21.5 ± 4.8
High FODMAPs	6.5 ± 2.3	2.3 ± 1.2*
Low‐FODMAPs percentage (%)	78.7 ± 4.0	90.1 ± 5.1*

*Note*: Based on the pictures sent back by the patients, we counted the total number of food types patients ate daily before and after low‐FODMAPs intervention.

* Compared to the predietary intervention, *p* < .05.

**TABLE 3:** Comparative analysis of dietary components in the low‐FODMAPs group and combined group at baseline and after dietary intervention (mean ± SD).

	Low‐FODMAPs group			Combined group		
	Baseline	4 weeks	*p*	Baseline	4 weeks	*p*
Energy, kcal/d	1919.3 ± 440.29	1656.4 ± 534.08	.104	1788.13 ± 465.67	1748.07 ± 678.36	.808
Protein, g/d	75.13 ± 16.32	74.38 ± 26.57	.934	69.24 ± 13.83	71.92 ± 31.38	.746
Fat, g/d	713.54 ± 1870.82	72.14 ± 23.25	.066	87.02 ± 32.75	76.54 ± 29.02	.302
Carbohydrates, g/d	215.46 ± 55.46	193.95 ± 103.95	.314	197.53 ± 54.76	201.51 ± 96.04	.911
Dietary fiber, g/d	13.41 ± 5.37	11.17 ± 5.08	.357	14.25 ± 3.60	13.44 ± 4.63	.690
Total FODMAP, g/d	2.81 ± 2.60	1.10 ± 1.32	.008*	1.71 ± 1.03	0.87 ± 0.45	.032*

* Compared to the baseline, *p* < .05.

### 3.2 Clinical outcomes

There were no significant differences in IBS‐SSS, abdominal pain, pain frequency, bloating, or bowel habits among the 4 groups at baseline (*p* > .05). After intervention, there was significant relief of IBS‐SSS in the low‐FODMAPs (37.5%, 44.2%), probiotics group (51.4%, 62.0%), and combined group (34.1%, 40.4%) at both the 2 weeks and 4 weeks, respectively, compared with the baseline in the corresponding same group (*p* < .05) (Table 4). At the 4 weeks, the IBS‐SSS was significantly improved in the low‐FODMAPs, probiotics group compared with the control group (*p* < .05), but not in the combined group (*p* > .05) (Table 4).

**TABLE 4:** Clinical outcomes of IBS patients after 4 weeks of intervention (mean ± SD).

Group	Weeks		
	Baseline	2 weeks	4 weeks
*Total score*			
Control	202.80 ± 90.97	179.60 ± 52.70	232.20 ± 113.74
Low‐FODMAPs	208.56 ± 57.26	130.83 ± 51.92*	116.00 ± 51.37*,**
Probiotic	254.63 ± 83.87	147.67 ± 61.99*	125.50 ± 67.46*,**
Combined	243.85 ± 78.79	172.45 ± 78.57*	159.30 ± 69.60*
*p*	.192	.187	.005
*Abdominal pain*			
Control	35.00 ± 13.69	25.00 ± 17.68	30.00 ± 27.37
Low‐FODMAPs	26.39 ± 21.82	11.11 ± 12.78*	9.72 ± 15.19*
Probiotic	32.29 ± 24.98	16.67 ± 15.93*	13.54 ± 16.45*
Combined	27.50 ± 21.31	23.75 ± 26.25	15.00 ± 12.57*
*p*	.711	.307	.210
*Pain frequency*			
Control	48.00 ± 48.17	64.00 ± 49.80	64.00 ± 49.80
Low‐FODMAPs	28.33 ± 33.08	10.56 ± 15.89*	8.33 ± 13.39*,**
Probiotic	35.42 ± 35.17	19.79 ± 23.75*	13.33 ± 21.65*,**
Combined	52.50 ± 39.55	33.00 ± 40.92*	26.75 ± 34.69*
*p*	.314	.094	.041
*Bloating*			
Control	30.00 ± 11.18	25.00 ± 17.68	45.00 ± 37.08
Low‐FODMAPs	31.67 ± 24.07	16.67 ± 12.13*	16.67 ± 14.85*
Probiotic	48.96 ± 30.82	23.96 ± 15.60*	21.88 ± 18.52*
Combined	33.75 ± 21.88	17.50 ± 18.32*	16.25 ± 16.77*
*p*	.163	.351	.170
*Bowel habits*			
Control	46.60 ± 38.11	33.20 ± 23.69	53.20 ± 30.02
Low‐FODMAPs	63.06 ± 22.71	50.06 ± 26.39*	48.11 ± 17.38*
Probiotic	68.13 ± 25.13	47.25 ± 22.04*	44.38 ± 19.10*
Combined	68.60 ± 25.14	51.60 ± 25.47*	53.25 ± 25.27*
*p*	.455	.535	.769
*Life impact*			
Control	33.20 ± 23.69	26.40 ± 14.76	40.00 ± 28.09
Low‐FODMAPs	57.44 ± 22.53	42.44 ± 15.67*	33.22 ± 28.08*
Probiotic	68.17 ± 20.89**	40.13 ± 17.20*	34.58 ± 20.93*
Combined	59.95 ± 25.76	46.60 ± 27.52*	46.55 ± 27.48*
*p*	.049	.287	.404

*Note*: *p* values indicate whether there is a significant difference among the four groups at different time points.

* Compared to the baseline in the corresponding same group, *p* < .05.

** Compared to the control group on the same date, *p* < .05.

Among the symptoms related to the IBS‐SSS, the grade, and frequency of abdominal pain, bloating, and the satisfaction of the bowel habits were significantly improved in the low‐FODMAPs, probiotics group, and combined group at both the 2 weeks and 4 weeks, compared with the baseline in the corresponding same group (*p* < .05) (Table 4). At the 4 weeks, the frequency of abdominal pain was significantly improved in the low‐FODMAPs, probiotics group compared with that in the control group (*p* < .05) (Table 4). Although there was improvement of the other symptoms, the grade of abdominal pain, bloating, and the satisfaction of the bowel habits had no statistical difference among the 4 groups at the 2 weeks and 4 weeks (*p* > .05) (Table 4).

According to the life impact, there were significant differences among the 4 groups at the baseline (*p* < .05) (Table 4). After 2 weeks and 4 weeks of intervention, the life impact was significantly improved as compared to the corresponding baseline group in the low‐FODMAP, probiotics, and combination group (*p* < .05) (Table 4), but no significant difference among the 4 groups at 2 weeks and 4 weeks (*p* > .05) (Table 4).

### 3.3 Microbiota outcomes

**FIGURE 2:**
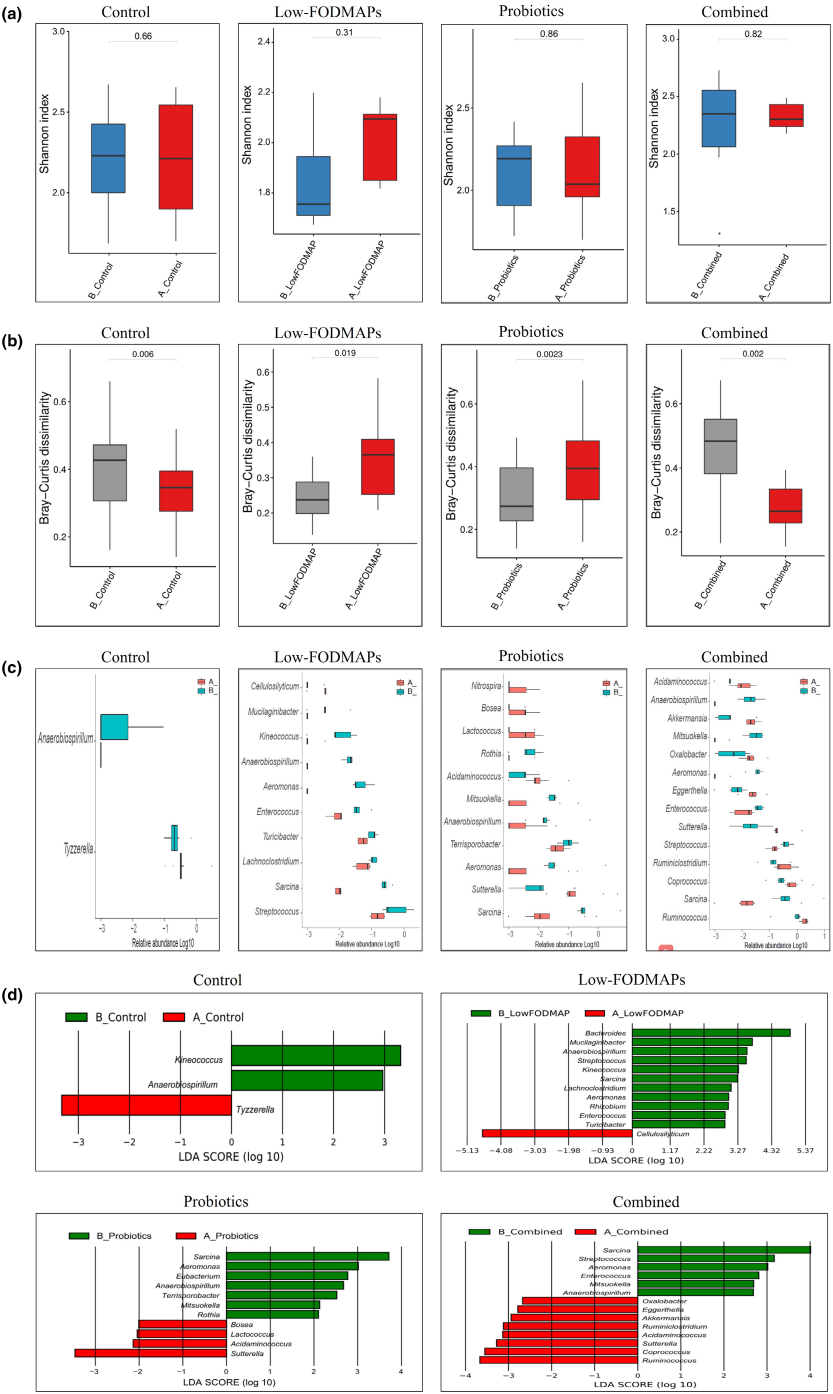
Altered gut microbiota profile in IBS patients at baseline and after 4 weeks of dietary and/or probiotic intervention. (a) Compared with baseline, there was no significant change in α‐diversity among the 4 groups (measured by the Shannon index). (b) The changes of β‐diversity in the 4 groups compared to each corresponding baseline (measured by Bray–Curtis dissimilarity). Bray–Curtis dissimilarity in the low‐FODMAPs and the probiotics group were significantly increased whereas that in the control and the combined group were significantly decreased, compared with baseline. (c) The significantly different microbiota at baseline and after 4 weeks of dietary and/or probiotic intervention in the 4 groups, histogram represented the relative abundance of microbiome in log 10 (showing the top 15 strains with mostly significant changes). (d) Linear discriminant effect size (LEFSe) changes visualized by LDA at baseline and after 4 weeks of dietary and/or probiotic intervention in the 4 groups (LDA >2, the length of bar chart shows the influence of taxa). A, after 4 weeks of dietary and/or probiotic intervention; B, baseline; IBS, irritable bowel syndrome; LDA, linear discriminant analysis.

There was no significant difference in operational taxonomic units (OTUs) or diversity among the 4 groups at baseline. After 4 weeks of follow‐up, significant differences in microbial signatures were observed. Compared to the baseline, there was no significant difference in α‐diversity between the control, low‐FODMAPs, probiotics, and combined groups, as measured by the Shannon index in Figure 2a (*p* > .05). The β‐diversity was significantly changed in the 4 groups. The Bray–Curtis dissimilarity in the low‐FODMAPs and probiotics groups was significantly increased, whereas that in the control and combined groups were significantly decreased (Figure 2b, *p* < .05). The composition of bacterial abundance was significantly changed in the 4 groups, and 15 strains with the most significant changes compared with the baseline were shown in Figure 2c. To analyze the bacterial community structure, linear discriminant analysis (LDA) was performed on the genera that met the LDA significance threshold greater than 2 (LDA >2, *p* < .05; Figure 2d). The abundance of Genus_*Bacteriodes*, *Mucilaginibacter*, *Anaerobiospirillum*, *Kineococcus*, *Sarcina*, *Lachnoclostridium*, etc. contributed to the difference before FODMAPs intervention whereas Genus_*Cellulosilyticum* was significantly enriched after the low‐FODMAPs intervention (Figure 2d). The abundance of *Sutterella*, *Acidaminococcus*, *Lactococcus*, and *Bosea* was significantly enriched after the supplementation of probiotics (Figure 2d). The combination of the low‐FODMAPs and probiotics significantly enriched the abundance of Genus_*Ruminococcus*, *Coprococcus*, *Sutterella*, *Acidaminococcus*, *Ruminiclostridium*, *Akkermansia*, *Eggerthella*, and *Oxalobacter* (Figure 2d).

### 3.4 Association analysis between intestinal microbiota and IBS symptoms

**FIGURE 3:**
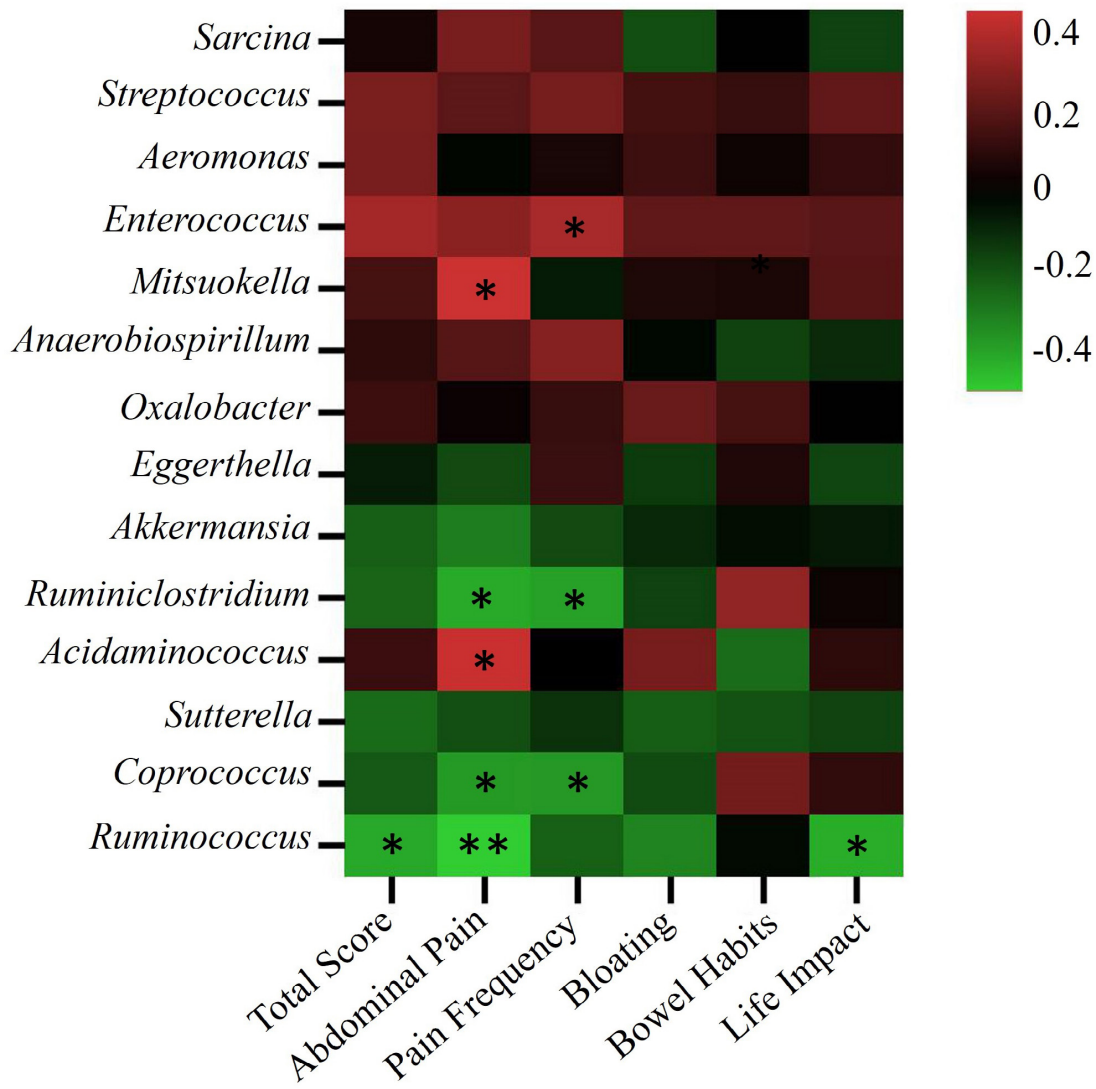
Association analysis between intestinal microbiota and IBS symptoms. **There is a significant correlation, at the .01 level (two‐sided). *Significant correlation at the .05 level (two‐sided).

We performed *Pearson* correlation analyses of gut microbiota and IBS symptoms (Figure 3). The abundance of *Ruminiclostridium*, *Coprococcus*, and *Ruminococcus* was found to be significantly negatively correlated with abdominal pain score (*p* < .05). However, the abundance of *Acidaminococcus* was significantly positively correlated with abdominal pain score (*p* < .05). The abundance of *Ruminiclostridium* and *Coprococcus* was significantly negatively correlated with the abdominal pain frequency score (*p* < .05). In addition, the abundance of *Ruminococcus* was negatively correlated with the IBS‐SSS total score and life impacts (*p* < .05).

### 3.5 SCFAs level

There was no significant difference in SCFA levels at 4 weeks, compared to baseline in the corresponding same group (Table S1).

## 4 DISCUSSION

This study reported that, compared to the conventional dietary, the low‐FODMAPs diet led to the relief of IBS‐SSS and alleviated the pain frequency after 4 weeks of intervention in Western China (Table 4). Compared to the corresponding baseline, the relief of IBS‐related symptoms including the alleviation of total severity score, abdominal pain, pain frequency, bloating, the satisfaction of bowel habits, and the life impact was more convincible after 2 weeks and 4 weeks of intervention. Although the combination of low‐FODMAPs and probiotics could not further alleviate IBS‐related symptoms, changes in the abundance of gut microbiota may be beneficial. Therefore, our study evaluated the effectiveness of low‐FODMAPs alone and the benefits of combination with probiotics for IBS patients in Western China.

The effectiveness of low‐FODMAPs in the clinic is closely related to the dietary background, which is generally treated as the control group in clinical trials. In the United Kingdom, a placebo‐controlled study comparing the conventional diet and low‐FODMAPs diet reported the alleviation of IBS‐SSS in the low‐FODMAPs group in patients with IBS (Staudacher et al., 2017). A randomized clinical trial from Thailand revealed that a low‐FODMAP diet significantly improved clinical symptom scores and reduced intestinal H_2_ production after 4 weeks compared with a commonly recommended diet in patients with moderate‐to‐severe IBS patients (Patcharatrakul et al., 2019). A follow‐up study of 18 IBS patients for 12 months found that long‐term personalized low‐FODMAPs diet can maintain the relief of gastrointestinal symptoms, improve quality of life, and sustain the abundance of fecal *Bifidobacterium* (Staudacher et al., 2022). An increasing number of studies have confirmed that a low‐FODMAPs diet can relieve the symptoms of IBS patients, but it is only effective for some patients. It has been found that the therapeutic effects of low‐FODMAPs diet combined with tryptophan restriction can be enhanced in IBS patients (Chojnacki et al., 2023). A low‐FODMAPs diet combined with β‐galacto‐oligosaccharides, a prebiotic, played a great role in relieving symptoms but did not reverse the decreased abundance of *Bifidobacterium* (Wilson et al., 2020). Therefore, although low‐FODMAPs dietary advice has been confirmed to be beneficial and recommended in the set of clinical IBS guidelines or reviews (Camilleri & Dilmaghani, 2023; Chey et al., 2022), most clinical trials were based on IBS patients with western diet.

Sichuan cuisine is one of the four major cuisines in China. The distinct secret of the cooking process is flavoring each ingredient separately, or in small groupings, and then combining them. To measure the content of FODMAP in the ingredients, we tried to detect the total content of FODMAPs in a set meal of Sichuan cuisine in our preliminary study. However, the FODMAPs content cannot be detected due to their complex composition. We evaluated the FODMAPs content in the ingredients through previous data (Biesiekierski et al., 2011; Chumpitazi et al., 2018; Muir et al., 2007, 2009; Prichard et al., 2016; Tuck et al., 2018; Varney et al., 2017). After dietary recommendation, the percentage of high FODMAPs was significantly decreased to 2.3%, whereas the percentage types of low‐FODMAPs was significantly increased to 90.1% (Table 2). FODMAP contents were significantly decreased compared to the baseline (*p* < .05, Table 3). Our study confirmed the effectiveness of low‐FODMAPs in providing dietary advice on the background of Chinese food in Western China, whether compared to the baseline or conventional dietary recommendations.

A recent systemic review, including 16 articles involving 777 patients with IBS, revealed relatively consistent changes in the fecal microbiota, namely, an increase in the ratio of *Firmicutes*: *Bacteroidetes* at the phylum level, and a decreased *Bacteroidia* and *Bacteroidales* at lower taxonomic levels (Duan et al., 2019). In our study, none of the groups showed changes in the abundance of *Firmicutes* or *Bacteroidetes* or the ratio of *Firmicutes* to *Bacteroidetes* at the phylum level. According to the LDA (>2) analysis, low‐FODMAPs significantly decreased the abundance of 11 genera, including *Bacteriodes*, *Mucilaginibacter*, *Anaerobiospirillum*, *Strepotcoccus*, *Kineococcus*, *Sarcina*, *Lachnoclostridium*, etc. *Bifidobacteria*, as the most popular decreased abundance after low‐FODMAPs intervention (Patcharatrakul et al., 2019; So et al., 2022), decreased in the low‐FODMAPs group. These data supported the regional characteristics of IBS and dietary intervention.

Interestingly, after the supplementation of multiple probiotics in our study, the abundance of genus_ *Ruminiclostridium, Coprococcus, Acidaminoccus, Ruminococcus, Sutterella*, etc. were significantly increased in the combined group. *Akkermansia muciniphila*, a specific genus dwelling in mucins, plays an important role in systemic metabolism (Depommier et al., 2019) and induces an intestinal adaptive immune response (Ansaldo et al., 2019). Increased abundance of *Akkermansia* is usually considered a healthy gut microbial community. *Ruminiclostridium cellulolyticum* and *Ruminococcus flavefaciens* are the model bacteria to produce cellulosomes and efficiently degrade cellulose from plant cells (Kampik et al., 2021; Yeoman et al., 2021), with significantly increased abundance, supporting the improvement of digestive function of plant fibers in the gut after combined intervention. *Sutterella* and *Acidaminoccu*s were the two consistently increased genus in both probiotics and combined group in our study. To our knowledge, the function and role of these two genera in IBS are still unclear, but a recent study reported that *Sutterella* has the capacity to degrade IgA (Kaakoush, 2020). *Coprococcus* is an important butyrate producer that plays an important role in reducing the intestinal inflammatory response and maintaining intestinal homeostasis (Singh et al., 2023; Yang et al., 2023). These changes were inconsistent with previous studies (Staudacher et al., 2017). Moreover, in our *Pearson* correlation analysis, *Ruminiclostridium*, *Coprococcus*, *Ruminococcus*, *Acidaminococcus*, *Ruminiclostridium*, and *Coprococcus* were significantly correlated with the improvement of IBS symptoms. Overall, the combination of a low‐FODMAPs diet and probiotics improved the gut microbiota in IBS patients, although improvement of IBS‐SSS was not observed compared to the low‐FODMAPs group.

SCFAs are produced by gut microbiota through the fermentation of nondigestible fibers and dietary carbohydrates, thus SCFAs' concentrations are closely related to diet types (Cong et al., 2022). The low‐FODMAPs diet was restricted by significantly reducing the types of high FODMAPs diet from 6 to 2, but not increasing the types of low‐FODMAPs diet in our study. Compared to the 30 types of food consumed every day, SCFAs may be more influenced by other types of food than by FODMAPs.

There were still several limitations of this study. First, we did not set the placebo group. One of our aims is to investigate whether the low‐FODMAPs diets‐induced alterations in the microbiota could be improved through the supplementation of probiotics. Considering that the placebo mainly affects subjective symptoms, but has a weak effect on objective indicators, such as changes in microbiota (Chahwan et al., 2019), we did not set a placebo in this study. We will consider designing the placebo in the control and Combined group in future studies. Second, patients with all three subtypes of IBS were included in our study. However, we could not distinguish the effect of the low‐FODMAPs dietary intervention on each subtype with the sample size in our study. Diarrhea‐predominant IBS patients have been preferred for the low‐FODMAPs in previous studies from other regions (Chojnacki et al., 2023; Y. Zhang et al., 2021). More than half of IBS patients in our study were in diarrhea subgroup. Therefore, we suggest that low‐FODMAPs diet may be more worth recommending in IBS diarrhea‐predominant patients. Thirdly, we did not screen people with lactose intolerance by methods such as hydrogen breath tests. Moreover, the changes in dietary intervention may affect other factors (e.g. intestinal motility), thus gut microbiota may be indirectly influenced by low‐FODMAPs. In addition, we only conducted a 4‐week study to demonstrate the short‐term efficacy of low‐FODMAPs diet. In recent meta‐analyses, we found that the 4‐week duration was used in most clinical studies of low‐FODMAPs intervention on IBS (Black et al., 2022; So et al., 2022; van Lanen et al., 2021). Finally, there is no significant difference in SCFA among the groups in our study. The sample size was calculated based on the remission rate of IBS‐SSS questionnaire. Therefore, the sample size cannot guarantee the statistical differences of SFCA, which manifested higher standard deviation in SCFA measurement in our study. More sample sizes will be needed in future work.

In conclusion, our study evaluated the effectiveness and safety of low‐FODMAPs in overall and specific IBS symptoms, based on the background of Chinese Western food. Low‐FODMAPs diet significantly improved the symptoms related to the IBS‐SSS and the IBS‐QOL and altered the composition of bacterial abundance. Furthermore, the combination of low‐FODMAPs and probiotics improved the changes in microbiota abundance after low‐FODMAPs intervention.

## AUTHOR CONTRIBUTIONS

**Yingying Liu:** Investigation (equal); writing – original draft (equal); writing – review and editing (equal). **Di Jin:** Formal analysis (equal); writing – review and editing (equal). **Tian He:** Formal analysis (equal). **Xinyi Liao:** Investigation (equal). **Limei Shao:** Investigation (equal). **Lei Shi:** Methodology (equal); project administration (equal); supervision (equal); writing – original draft (equal). **Ling Liu:** Funding acquisition (equal); methodology (equal); project administration (equal); writing – original draft (equal); writing – review and editing (equal).

## FUNDING INFORMATION

Our study was funded by the General Program of National Natural Science Foundation of China (NSFC) (81970463) and the Regional Innovation Cooperation Program, Science and Technology Department of Sichuan Province (2022YFQ0053).

## CONFLICT OF INTEREST STATEMENT

None.

## Supporting information


Table S1.

Data S1.

## Data Availability

The data that support the findings of this study are available from the corresponding author upon reasonable request.
