# Supplementary material for: Effect of the combined intervention of low‐FODMAPs diet and probiotics on IBS symptoms in Western China: A randomized controlled trial
Source: Food Sci Nutr. 2024 Feb 29;12(6):3993–4004. doi: 10.1002/fsn3.4057 (PMC11167163; doi:10.1002/fsn3.4057)
Supplement: Supplementary file 1 — Table S1. [file FSN3-12-3993-s001.docx]

**Supplementary Table 1**. SCFAs of IBS patients after 4 weeks intervention(mean ± SD)

|  | Groups | Baseline | 4 weeks |  |
| --- | --- | --- | --- | --- |
| Acetic acid | Control | 1593.09±860.93 | 1525.50±930.25 |  |
|  | Low-FODMAPs | 926.23±489.50 | 954.22±517.88 |  |
|  | Probiotic | 1584.57±960.12 | 1443.78±653.24 |  |
|  | Combined | 1836±1483.17 | 1283.87±464.88 |  |
| Propionic acid | Control | 623.84±383.47 | 518.67±295.76 |  |
|  | Low-FODMAPs | 434.07±211.57 | 377.52±294.50 |  |
|  | Probiotic | 576.25±542.52 | 562.46±397.80 |  |
|  | Combined | 990.08±1011.01 | 416.41±167.37 |  |
| Isobutyric acid | Control | 132.53±62.67 | 113.27±53.04 |  |
|  | Low-FODMAPs | 70.75±16.51 | 71.43±12.89 |  |
|  | Probiotic | 94.15±17.26 | 376.43±726.53 |  |
|  | Combined | 304.99±367.67 | 142.02±88.89 |  |
| Butyric acid | Control | 2353.92±1443.13 | 2170.87±1447.40 |  |
|  | Low-FODMAPs | 1166.22±523.28 | 1424.46±1035.45 |  |
|  | Probiotic | 2031.68±2236.52 | 2066.69±749.68 |  |
|  | Combined | 1913.26±1319.18 | 1918.53±1019.24 |  |
| Isovaleric acid | Control | 346.53±224.51 | 347.06±234.95 |  |
|  | Low-FODMAPs | 121.81±38.93 | 278.97±253.78 |  |
|  | Probiotic | 344.15±478.57 | 310.62±122.04 |  |
|  | Combined | 408.96±284.14 | 284.54±309.70 |  |
| Valeric acid | Control | 13.86±7.59 | 14.52±7.74 |  |
|  | Low-FODMAPs | 7.83±0.93 | 9.15±2.05 |  |
|  | Probiotic | 19.38±24.73 | 41.48±80.38 |  |
|  | Combined | 18.30±13.91 | 14.91±10.51 |  |
|  |  |  |  |  |
