# Supplementary material for: Effect of the combined intervention of low‐FODMAPs diet and probiotics on IBS symptoms in Western China: A randomized controlled trial
Source: Food Sci Nutr. 2024 Feb 29;12(6):3993–4004. doi: 10.1002/fsn3.4057 (PMC11167163; doi:10.1002/fsn3.4057)
Supplement: Supplementary file 2 — Data S1. [file FSN3-12-3993-s002.docx]

**Supplementary Materials**

**Irritable bowel Syndrome(IBS) questionnaire**

Name:

Age:

Address:

Telephone:

Marital status: Single/ Married/ Divorced/ Widowed/Co-habit

Occupation:

Sec: Male/ Female

Nation:

**INSTRUCTIONS**

This form is designed to record and monitor the severity of your IBS. It is to be expected that your symptoms might vary over time, so please try and answer the questions based on how you currently feel (is over the last 10 days or so). All information will be kept in strict confidence.

**Part 1**: Severity score

1. A. Do you currently suffer from abdominal (tummy) pain?

□ YES □ No1.

B. If yes, how severe is your abdominal (tummy) pain?

□ no pain □ not very severe □ quiet severe □ severe □ very severe

C. Please enter the number of days that you get the pain in every 10 days .

For example if you enter 4 it means that you get pain 4 out of 10 days. If you get pain every day enter 10 .

Number of days with pain:_____x10

2. A. Do you currently suffer from abdominal distension?

□ YES □ No1.

B. If yes, how sever is your abdominal distension/tightness?

□ no distension □ not very severe □ quiet severe □ severe □ very severe

C. Please enter the number of days that you get abdominal distension/tightness in every 10 days .

Number of days with abdominal distension/tightness :_____x10

3. How satisfied are you with your bowel habit?

□ very happy □ quiet happy □ unhappy □ very unhappy

4. How much your Irritable Bowel Syndrome is affecting or interfering with your life in general?

□ not at all □ not much □ quiet a lot □ completely

IBS SEVERITY SCORE : _____

**Part 2**: Other IBS data

5. A. What is the most number of times you open your bowels per day/ week/ month?

Number of times: ____ per day/ week/ month

B. What is the least number of times you open your bowels per day/ week/ month?

Number of times: ____ per day/ week/ month

6. In the following question you may circle more than one answer:

Are you motions ever:

a) nomal often/ occasionally/ never

b) hard often/ occasionally/ never

c) very thin (like string) often/ occasionally/ never

d) in small pieces (like rabbit pellets) often/ occasionally/ never

e) mushy (like porridge) often/ occasionally/ never

f) watery often/ occasionally/ never

7. In the following questions you may circle more than one answer:

Do you ever:

a) pass mucus (or slime of jelly) with your motions □YES □NO

b) pass blood with your motions □YES □NO

c) have a hurry/rush to the toilet to open your bowels □YES □NO

d) strain to open your bowels □YES □NO

e) feel you haven’t emptied your bowel completely □YES □NO

after you have passed a motion.

Please mark with a cross(X) on the diagram below where you get your pain (use more than one X if necessary)


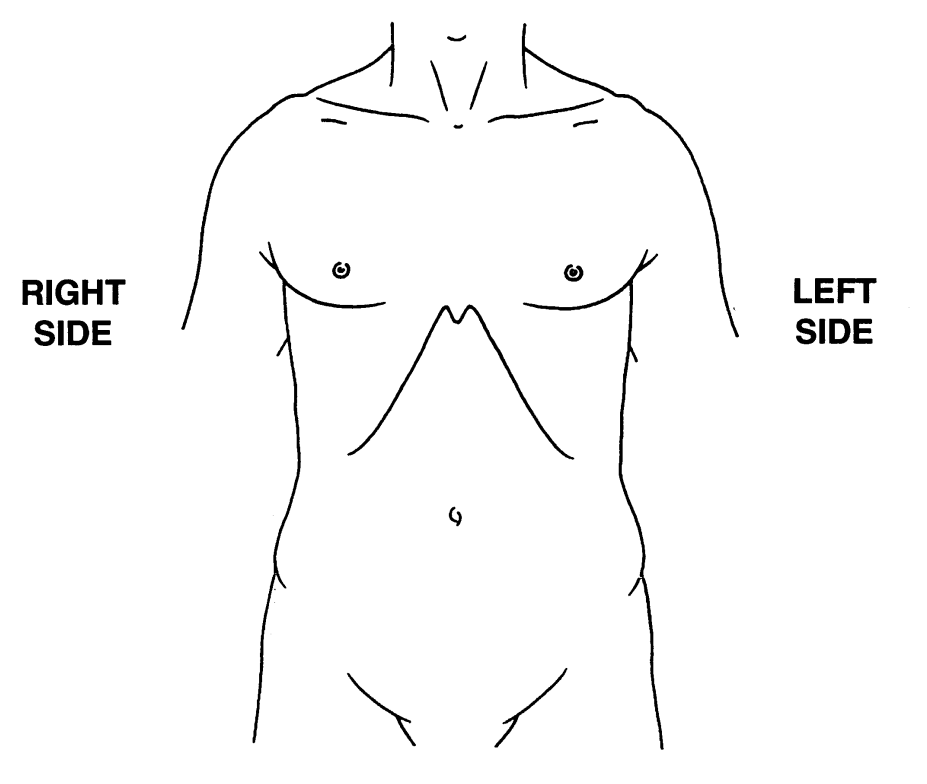


8. Do you ever:

A. notice your stools are more frequent or loose when you get pain

□YES □NO

B. notice whether the pain is frequently eased by opening your bowels

□YES □NO

9. In the last year on approximately how many weeks were you:

A. absent from work due to IBS ________weeks

B. at work suffering from IBS ________weeks

**Part 3**: Irritable Bowel Syndrome Quality of Life (IBS-QOL) questionnaire

Items 1, 2, 4, 8 10, 12, 13, 16, 25-29, and 34 use the following response scale:

1=not at all

2=slightly

3=moderately

4=quite a bit

5=extremely

Items 3, 5±7, 11, 14, 15, 17-24, and 30-33 use the following response scale:

1=not at all

2=slightly

3=moderately

4=quite a bit

5=a great deal

Q1. I feel helpless because of my bowel problems.

Q2. I am embarrassed by the smell caused by my bowel problems.

Q3. I am bothered by how much time I spend on the toilet.

Q4. I feel vulnerable to other illnesses because of my bowel problems.

Q5. I feel fat because of my bowel problems.

Q6. I feel like I’m losing control of my life because of my bowel problems.

Q7. I feel my life is le ss enjoyable because of my bowel problems.

Q8. I feel uncomfortable when I talk about my bowel problems.

Q9. I feel depressed about my bowel problems.

Q10. I feel isolated from others because of my bowel problems.

Q11. I have to watch the amount of food I eat because of my bowel problems.

Q12. Because of my bowel problems, sexual activity is difficult for me.

Q13. I feel angry that I have bowel problems.

Q14. I feel like I irritate others be cause of my bowel problems.

Q15. I worry that my bowel problems will get worse .

Q16. I feel irritable because of my bowel problems.

Q17. I worry that people think I exaggerate my bowel problems.

Q18. I feel I get less done because of my bowel problems.

Q19. I have to avoid stressful situations because of my bowel problems.

Q20. My bowel problems reduce my sexual de sire.

Q21. My bowel problems limit what I can wear.

Q22. I have to avoid strenuous activity because of my bowel problems.

Q23. I have to watch the kind of food I eat be cause of my bowel problems.

Q24. Because of my bowel problems, I have difficulty being around people I do not know well.

Q25. I feel sluggish because of my bowel problems.

Q26. I feel uncle an because of my bowel problems.

Q27. Long trips are difficult for me because of my bowel problems.

Q28. I feel frustrated that I cannot eat when I want because of my bowel problems.

Q29. It is important to be near a toile t because of my bowel problems.

Q30. My life revolve s around my bowel problems.

Q31. I worry about losing control of my bowels.

Q32. I fear that I won’t be able to have a bowel movement.

Q33. My bowel problems are affecting my closest relationships.

Q34. I feel that no one understands my bowel problems.

**Informed consent**

**四川大学 华西医院**

基于中国传统饮食的低FODMAP 饮食联合益生菌

治疗肠易激综合征的临床疗效观察及机制探讨

知情同意书·知情告知页

亲爱的患者：

您已经确诊为肠易激惹综合征。我们将邀请您参加基于中国传统饮食的低FODMAP 饮食联合益生菌治疗肠易激综合征的临床疗效观察及机制探讨，课题编号： 2022YFQ0053 。本研究方案已经得到 中国注册临床试验伦理审查委员会 审核，同意进行临床研究。

在您决定是否参加这项研究之前，请尽可能仔细阅读以下内容。它可以帮助您了解该项研究目的及其意义、研究的程序和期限、参加研究后可能给您带来的益处、风险和不适。如果您愿意，你需要配合所做的检查和医师所要求的其它事项，您也可以和您的亲属、朋友一起讨论，或者请医生给予解释，帮助您做出决定。

1. **研究目的及意义**

肠易激综合征(Irritable bowel syndrome，IBS) 是一组持续或间歇发作，以腹痛、腹胀、排便习惯和（或）大便性状改变为临床表现，而缺乏胃肠道结构和生化异常的肠道功能紊乱性疾病。IBS是临床消化科医师面临的最常见胃肠道疾病，发病率逐年增加，现有的药物治疗效果并不理想。IBS严重影响患者的生活质量，带来巨大的经济和精神负担。由于IBS的发病与食物不耐受及肠道微生态失衡相关，所以，恰当的饮食调整及益生菌治疗均被认为是IBS的新型治疗方案，受到人们高度关注。

国外的新近研究认为，以控制多种食物为基础的低发酵性寡糖、双糖、单糖及多元醇（Fermentable oligosaccharides, disaccharides and monosaccharides and polyols，FODMAP）饮食对IBS治疗的有效性。FODMAP主要富含乳糖（如乳制品、生奶酪等）、果糖（如蜂蜜、苹果等）、果聚糖（如芦笋、甜菜根等）和多元醇（如山梨糖醇、甘露糖醇等）等碳水化合物。FODMAP饮食的共同特点为不易被小肠吸收，升高肠腔渗透压，在结直肠中易被发酵产气，从而引起腹痛、腹胀、腹部不适等IBS症状。低FODMAP饮食广泛应用在澳大利亚、新西兰、欧洲和北美一些地区IBS的管理。

尽管低FODMAP饮食逐渐在IBS临床治疗中推广，但这些研究均来源于基于西方饮食（包括澳大利亚、挪威、丹麦、新西兰、瑞士、瑞典及英国）为背景的临床观察，基于中国传统饮食为基础的低FODMAP饮食对IBS的疗效未见报道。另一方面，长期控制FODMAP 摄入对人体具有潜在的健康隐患。低FODMAP饮食主要表现为影响肠道菌群的数量及/或种类，存在潜在的健康隐患，从而影响FODMAP饮食的安全使用。益生菌制剂可通过改善宿主肠道微生态而改善IBS患者的总体症状，已被国内外广泛应用于临床IBS的治疗。因此我们原创性提出基于中国传统饮食背景下，低FODMAP饮食联合益生菌治疗IBS的方案，拟通过一项单中心随机对照研究，观察该治疗方案对IBS患者临床症状、生活质量及精神状况的疗效及安全性，并测定它们对肠道微生态菌群、粪便短链脂肪酸水平的影响。该研究可能会为IBS患者缓解临床症状、改善生活质量提供更好、更有效的治疗方案；同时可能筛选出有效治疗IBS的肠道微生态菌株和短链脂肪酸分子，试图使您受益。

1. **哪些人不宜参加研究**
2. 伴发其他器质性疾病（如糖尿病、心、肺功能不全、腹部其它器质性疾病）；2）腹腔手术病史；3）孕妇、哺乳期妇女；4）既往接受过临床营养师管理的患者；5）正参加其它临床试验的患者；6）研究人员认为其他原因不适合临床试验者。

**三、如果参加研究将需要做什么？**

1. 在您入选研究前，医生将询问、记录您的病史，并记录您的检查结果。

您将完成问卷调查、生化检测、腹部B超、胃镜及结肠镜筛查排除全身及局部器质性疾病。根据结果确定方案，包括可否进入试验等。

若您是合格的纳入者，您可自愿参加研究，签署知情同意书，遵医嘱治疗，收集粪便完成肠道微生态及短链脂肪酸水平检测。

如您不愿参加研究，我们将按您的意愿施治。

2. 若您自愿参加研究，将按以下步骤进行：

医生将向交代研究的目的、过程和注意事项；

您需要在或到医院进行检查和随访的时间、次数及注意事项。

3. 需要您配合的其他事项

您必须按医生和您约定的随访时间到来医院就诊（随访阶段，医生可能通过电话、微信、登门的方式了解您的情况）。

在研究期间您需要遵医嘱治疗。如您需要进行相关治疗，请事先与您的医生取得联系。

**四、参加研究可能的获益的是什么？**

1. IBS病情迁延，通过饮食调控可长期、安全的控制IBS相关症状，改善您的治疗效果，降低死亡风险，减少治疗费用。
2. 益生菌广泛应用于IBS研究，我科在肠道疾病治疗方面有丰富的先进诊疗经验，营养科可提供具体的营养咨询，各领域专家参与该研究，整个医疗团队将为您提供最优治疗方案及医疗服务。

**五 、参加研究可能出现的不良反应、风险和不适 是什么？**

1. 可能影响肠道菌群的数量及/或种类
2. 可能带来营养不良的风险

**六、参加研究的安全保障**

国外的新近研究认为，西方饮食背景下，以控制多种食物为基础的低FODMAP饮食对IBS治疗的有效性，但是，低FODMAP饮食影响肠道菌群的数量及/或种类，存在潜在的健康隐患，从而影响FODMAP饮食的安全使用。如果出现上述不良情况，您将得到积极治疗。

参加本研究的患者将根据随机原则，分为接受正常、低FODMAP、益生菌、低FODMAP+益生菌治疗。不论您在任何时候病情加重，医生均将及时采取最好的治疗方案进行治疗。

四川大学华西医院是中国国家级诊疗中心、国家药物临床试验机构，医疗水平处于全国先进行列。本研究的治疗团队的专家小组成员均是在肠道疾病的诊断及治疗、营养治疗方面有资深经验的医师。本研究方案及知情同意书均经过代表患者利益的伦理委员会的审核批准。受试者的权利、安全、健康是高于科学和社会利益而予以优先考虑。

**七、有关费用**

本研究中对肠道微生态菌群、粪便短链脂肪酸检测不收取任何费用。

如果您在研究过程中出现任何不适时，可与本研究小组联系。如果经医学专家委员会鉴定其与研究有关，我们将按照我国《药物临床试验质量管理规范》的规定对与研究相关的损害提供治疗的费用及相应的经济补偿。

对于您同时合并的其他疾病所需的治疗和检查，将不在免费的范围之内。

**八、个人信息的保密和标本处理**

您的医疗记录（研究病历/CRF、化验单等）将完整地保存在您所就诊的医院。研究者、伦理委员会和药品监督管理部门将被允许查阅您的医疗记录。任何有关本项研究结果的公开报告将不会披露您的个人身份。我们将在法律允许的范围内，尽一切努力保护您个人医疗资料的隐私。在整个研究过程中，您的粪便只用于科学研究。这些标本在研究结束后，将按照医院相关规定统一销毁。

**九、怎样获得更多的信息？**

您可以在任何时间提出有关本项研究的任何问题，并得到相应的解答。

如果在研究过程中有任何重要的新信息，可能影响您继续参加研究的意愿时，您的医生将会及时通知您。

**十、可以自愿选择参加研究和中途退出研究**

是否参加研究完全取决于您的意愿。您可以拒绝参加此项研究，或在研究过程中的任何阶段退出本研究，这都不会影响您和医生间的关系，都不会影响对您的医疗或有其他方面利益的损失。

出于对您的最大利益考虑，医生或研究者可能会在研究过程中随时中止您继续参加本项研究。

如果您因为任何原因从研究中退出，您可能被询问有关情况。如果医生认为需要，您也可能被要求进行实验室检查和体格检查。

此外，如发生以下情况将退出或中止研究：患者因不能按要求完成相关检查，影响数据采集；出现其他疾病或严重并发症而需要治疗或抢救，可能对本研究方案造成影响；发生不可接受的不良事件；没有遵循方案进行研究；患者错误入选；患者撤回知情同意书。

**十一、是否参加？**

是否参加本项研究由您自己（和您的家人）决定。

在您做出参加研究的决定前，请尽可能向你的医生询问有关问题。

感谢您阅读以上材料。如果您决定参加本项研究，请告诉您的医生，他/她会为您安排一切有关研究的事务。请您保留这份资料。

知情同意书.同意签字页

**临床研究项目名称：**基于中国传统饮食的低FODMAP 饮食联合益生菌治疗肠易激综合征的临床疗效观察及机制探讨

**课题承担单位: 四川大学华西医院**

**课题协作单位： 无**

**课题任务书编号： 2022YFQ0053**

**同意声明**

我已经阅读了上述有关本研究的介绍，而且有机会就此项研究与医生讨论并提出问题。我提出的所有问题都得到了满意的答复。

我知道参加本研究可能产生的风险和受益。我知晓参加研究是自愿的，我确认已有充足时间对此进行考虑，而且明白：

- 我可以随时向医生咨询更多的信息。
- 我可以随时退出本研究，而不会受到歧视或报复，医疗待遇与权益不会受到影响。
- 我同意医生收集我的粪便标本，对其进行检测研究，并在研究完成后对标本进行合理的处置。
- 如果因病情变化我需要采取任何其他的治疗，我会在事先征求医生的意见，或在事后如实告诉医生。
- 我同意伦理委员会查阅我的研究资料。
- 我将获得一份经过签名并注明日期的知情同意书副本。

最后，我决定同意参加本项研究，并保证尽量遵从医嘱。

患者签名： ＿ ＿ ＿ ＿ 年 ＿ ＿ 月 ＿ ＿ 日

联系电话：

我确认已向患者解释了本试验的详细情况，包括其权力以及可能的受益和风险，并给其一份签署过的知情同意书副本。

医生签名： 　 ＿ ＿ ＿ ＿ 年 ＿ ＿ 月 ＿ ＿ 日

医生的工作电话：


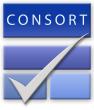
CONSORT 2010 checklist of information to include when reporting a randomised trial*

| Section/Topic | Item No | Checklist item | Reported on page No |
| --- | --- | --- | --- |
| Title and abstract | | | |
|  | 1a | Identification as a randomised trial in the title | 1 |
|  | 1b | Structured summary of trial design, methods, results, and conclusions (for specific guidance see CONSORT for abstracts) | 2 |
| Introduction | | | |
| Background and objectives | 2a | Scientific background and explanation of rationale | 3-5 |
|  | 2b | Specific objectives or hypotheses | 5 |
| Methods | | | |
| Trial design | 3a | Description of trial design (such as parallel, factorial) including allocation ratio | 5-9 |
|  | 3b | Important changes to methods after trial commencement (such as eligibility criteria), with reasons | 7-9 |
| Participants | 4a | Eligibility criteria for participants | 6 |
|  | 4b | Settings and locations where the data were collected | 5-6 |
| Interventions | 5 | The interventions for each group with sufficient details to allow replication, including how and when they were actually administered | 6-9 |
| Outcomes | 6a | Completely defined pre-specified primary and secondary outcome measures, including how and when they were assessed | 10 |
|  | 6b | Any changes to trial outcomes after the trial commenced, with reasons |  |
| Sample size | 7a | How sample size was determined | 10-11 |
|  | 7b | When applicable, explanation of any interim analyses and stopping guidelines | 6 |
| Randomisation: |  |  |  |
| Sequence generation | 8a | Method used to generate the random allocation sequence | 6 |
|  | 8b | Type of randomisation; details of any restriction (such as blocking and block size) | 6 |
| Allocation concealment mechanism | 9 | Mechanism used to implement the random allocation sequence (such as sequentially numbered containers), describing any steps taken to conceal the sequence until interventions were assigned | 6 |
| Implementation | 10 | Who generated the random allocation sequence, who enrolled participants, and who assigned participants to interventions | 6 |
| Blinding | 11a | If done, who was blinded after assignment to interventions (for example, participants, care providers, those assessing outcomes) and how | 6 |
|  | 11b | If relevant, description of the similarity of interventions |  |
| Statistical methods | 12a | Statistical methods used to compare groups for primary and secondary outcomes | 10-11 |
|  | 12b | Methods for additional analyses, such as subgroup analyses and adjusted analyses | 10-11 |
| Results | | | |
| Participant flow (a diagram is strongly recommended) | 13a | For each group, the numbers of participants who were randomly assigned, received intended treatment, and were analysed for the primary outcome | 11 |
|  | 13b | For each group, losses and exclusions after randomisation, together with reasons | 11 |
| Recruitment | 14a | Dates defining the periods of recruitment and follow-up | 11 |
|  | 14b | Why the trial ended or was stopped |  |
| Baseline data | 15 | A table showing baseline demographic and clinical characteristics for each group | 11 |
| Numbers analysed | 16 | For each group, number of participants (denominator) included in each analysis and whether the analysis was by original assigned groups | 11 |
| Outcomes and estimation | 17a | For each primary and secondary outcome, results for each group, and the estimated effect size and its precision (such as 95% confidence interval) | 8-11 |
|  | 17b | For binary outcomes, presentation of both absolute and relative effect sizes is recommended | 9-13 |
| Ancillary analyses | 18 | Results of any other analyses performed, including subgroup analyses and adjusted analyses, distinguishing pre-specified from exploratory | 9-13 |
| Harms | 19 | All important harms or unintended effect in each group (for specific guidance see CONSORT for harms) |  |
| Discussion | | | |
| Limitations | 20 | Trial limitations, addressing sources of potential bias, imprecision, and, if relevant, multiplicity of analyses | 10-21 |
| Generalisability | 21 | Generalisability (external validity, applicability) of the trial findings | 19 |
| Interpretation | 22 | Interpretation consistent with results, balancing benefits and harms, and considering other relevant evidence | 13-19 |
| Other information | | |  |
| Registration | 23 | Registration number and name of trial registry | 5 |
| Protocol | 24 | Where the full trial protocol can be accessed, if available |  |
| Funding | 25 | Sources of funding and other support (such as supply of drugs), role of funders | 21 |

*We strongly recommend reading this statement in conjunction with the CONSORT 2010 Explanation and Elaboration for important clarifications on all the items. If relevant, we also recommend reading CONSORT extensions for cluster randomised trials, non-inferiority and equivalence trials, non-pharmacological treatments, herbal interventions, and pragmatic trials. Additional extensions are forthcoming: for those and for up to date references relevant to this checklist, see [www.consort-statement.org](http://www.consort-statement.org).
